# Prognostic Impact of Jab1, p16, p21, p62, Ki67 and Skp2 in Soft Tissue Sarcomas

**DOI:** 10.1371/journal.pone.0047068

**Published:** 2012-10-05

**Authors:** Sveinung W. Sorbye, Thomas K. Kilvaer, Andrej Valkov, Tom Donnem, Eivind Smeland, Khalid Al-Shibli, Roy M. Bremnes, Lill-Tove Busund

**Affiliations:** 1 Department of Clinical Pathology, University Hospital of North Norway, Tromso, Norway; 2 Institute of Medical Biology, University of Tromso, Tromso, Norway; 3 Department of Oncology, University Hospital of North Norway, Tromso, Norway; 4 Institute of Clinical Medicine, University of Tromso, Tromso, Norway; 5 Department of Pathology, Nordland Central Hospital, Bodo, Norway; Ospedale Pediatrico Bambino Gesù, Italy

## Abstract

**Purpose:**

The purpose of this study is to clarify the prognostic significance of expression of Jab1, p16, p21, p62, Ki67 and Skp2 in soft tissue sarcomas (STS). Optimised treatment of STS requires better identification of high risk patients who will benefit from adjuvant therapy. The prognostic significance of Jab1, p16, p21, p62, Ki67 and Skp2 in STS has not been sufficiently investigated.

**Experimental Design:**

Tissue microarrays from 193 STS patients were constructed from duplicate cores of viable and representative neoplastic tumor areas. Immunohistochemistry was used to evaluate the expression of Jab1, p16, p21, p62, Ki67 and Skp2.

**Results:**

In univariate analyses, high tumor expression of Ki67 (P = 0.007) and Skp2 (P = 0.050) correlated with shorter disease-specific survival (DSS). In subgroup analysis, a correlation between Skp2 and DSS was seen in patients with malignancy grade 1 or 2 (P = 0.027), tumor size >5 cm (P = 0.018), no radiotherapy given (P = 0.029) and no chemotherapy given (P = 0.017). No such relationship was apparent for Jab1, p16, p21 and p62; but p62 showed a positive correlation to malignancy grade (P = 0.019). Ki67 was strongly positively correlated to malignancy grade (P = 0.001). In multivariate analyses, Skp2 was an independent negative prognostic factor for DSS in women (P = 0.009) and in patients without administered chemotherapy or radiotherapy (P = 0.026).

**Conclusions:**

Increased expression of Skp2 in patients with soft tissue sarcomas is an independent negative prognostic factor for disease-specific survival in women and in patients not administered chemotherapy or radiotherapy. Besides, further studies are warranted to explore if adjuvant chemotherapy or radiotherapy improve the poor prognosis of STS with high Skp2 expression.

## Introduction

Soft tissue sarcomas (STS) are a heterogeneous and highly malignant group of tumors originating from mesenchymal lineage. Local recurrence is common (20%) and metastases occur in one third of patients [1]. Prognostic markers in potentially curable STS should guide therapy after surgical resection. Neoadjuvant therapy is increasingly used and may improve prognosis in high-risk cases [2], but requires prognostic factors that can be evaluated preoperatively. Currently used prognostic factors mainly include clinicopathological variables such as tumor type, size, depth, malignancy grade, necrosis, vascular invasion, and growth pattern, which are combined into different prognostic systems [3]–[9].

The loss of cell cycle control is a critical step in the development of neoplasia. The cell cycle is a series of carefully coordinated and regulated steps that govern cellular proliferation. Cyclin-dependent kinases (CDK) phosphorylate the retinoblastoma (Rb) protein, a classic tumor suppressor and key component of the G1/S checkpoint. This allows DNA replication to proceed. Inhibitors of CDK, such as p16^INK4A^, p21, and p27 act as brakes on progression through the cell cycle.

The human Jun activation domain binding protein 1 (Jab1) was originally identified as a coactivator of the gene regulatory AP-1 proteins (Jun/Fos protooncogenes) involved in the control of cell proliferation [10]. Jab1 directly binds to p27 and induces nuclear export and subsequent degradation [11]. Some studies indicated that Jab1 can interact specifically with the protein form of the CDK inhibitor 27 and shuttle p27 from the nucleus to the cytoplasm. And further to decrease the cellular amount of p27 by accelerating p27 degradation via the ubiquitin-proteasome system [12], [13]. Other reports have shown that overexpression of Jab1 and low expression of p27 is associated with more advanced tumor stage and poor prognosis in several human cancers [11], [14]–[16].

**Figure 1:**
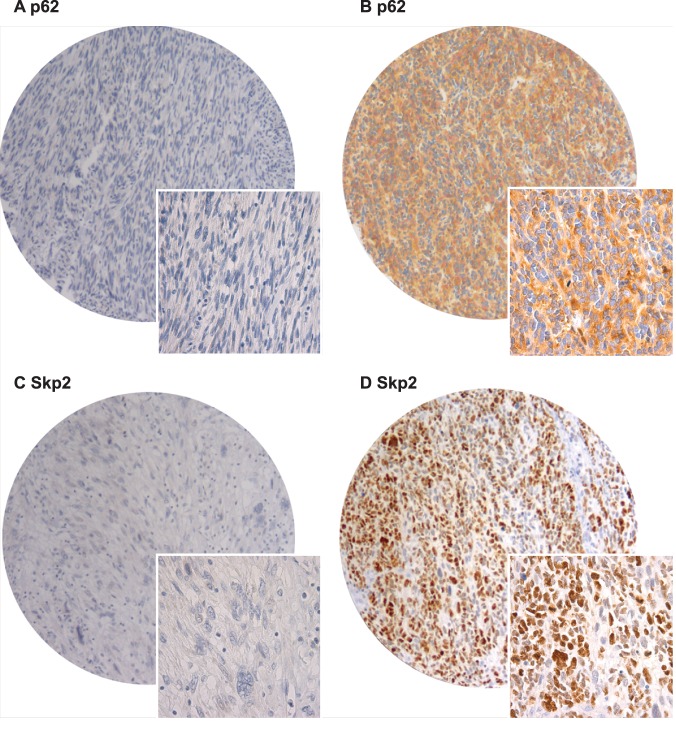
Immunohistochemistry microscopic pictures of tissue micro array of soft tissue sarcoma representing different expression of p62 and Skp2. (A) p62 low score; (B) p62 high score; (C) Skp2 low score; (D) Skp2 high score. Original magnification × 400.

The CDK inhibitor p16^INK4a^ (p16) protein belongs to the INK4 family of CDK inhibitors [17]. CDK inhibitors are negative regulators of the process of pRb hyperphosphorylation. The INK4 family of CDK inhibitors binds to CDK4/6 and the D family of cyclins to prevent formation of the cyclin–CDK complex required to phosphorylate pRb [17]. p16 has been identified as a tumor suppressor [18]. The gene encoding p16 is deleted in a high percentage of malignant cell lines and tissues [19]–[21]. p16 is important in cell senescence, and some studies have identified a role for p16 in cell proliferation and angiogenesis [22], [23]. A model of murine rhabdomyosarcoma has been produced through a subsequent genetic manipulations, among others p16 deletion [24], [25]. Still, the role of cell cycle regulators in the genesis of mesenchymal neoplasia is less well studied and the role of the p16 protein in STS has not been sufficiently investigated.

p21 (Waf1) is a cell cycle regulator, implicated in a variety of pathways [26]. The product of the CDKN1A gene (p21) binds to and inhibits the activity of CDK2/4 complexes, and thus functions as a regulator of cell cycle progression at the G1 checkpoint. Ki67 is involved in the synthesis of ribosomes and appears to be a necessary requirement for cell proliferation [27].

The intricate signalling network that determines whether cells grow, undergo senesce or die, achieves a remarkable degree of specificity with a relatively small number of signalling molecules [28]. Studies employing knockout, transgenic, and knockin mice have shown that p62 plays critical roles in a number of cellular functions, including bone remodelling, obesity, and cancer [29]–[31].

S-phase kinase-associated protein 2 (Skp2) is a member of mammalian F-box proteins, which displays S-phase-promoting function through ubiquitin-mediated proteolysis of the CDK inhibitor p27. Skp2 has been shown to regulate cellular proliferation by targeting several cell cycle-regulated proteins for ubiquitination and degradation. Skp2 has also been demonstrated to display an oncogenic function since its overexpression has been observed in many human cancers [32].

The purpose of this study was to clarify the prognostic significance of Jab1, p16, p21, p62, Ki67 and Skp2 expression in non-gastrointestinal stromal tumor (non-GIST) STS. To achieve this, we analyzed the expression of these markers in 193 patients with non-GIST STS in relation to demographic and other clinicopathological variables.

**Table 1:** Prognostic clinicopathological variables as predictors for disease-specific survival of soft tissue sarcomas (univariate analysis, log rank test), N = 193.

Characteristic	Patients(n)	Patients(%)	Median survival(months)	5-Year survival(%)	P
**Age**					
≤20 years	17	9	190	47	0.064
21–60 years	85	44	235	63	
>60 years	91	47	111	51	
**Gender**					
Male	81	42	235	60	0.087
Female	112	58	180	53	
**Nationality**					
Norwegian	131	68	228	62	0.005
Russian	62	32	81	44	
**Histology**					
Pleomorphic sarcoma	57	30	52	45	0.031
Leiomyosarcoma	47	24	89	64	
Liposarcoma	32	17	NR	71	
MF/MFT	16	8	123	56	
Angiosarcoma	8	4	10	38	
Rhabdomyosarcoma	9	5	NR	67	
MPNST	9	5	NR	56	
Synovial sarcoma	12	6	31	30	
Other STS	3	2	NR	–	
**Tumor localization**					
Extremities	78	40	201	56	0.922
Trunk	37	19	214	53	
Retroperitoneum	27	14	135	51	
Head/Neck	13	7	191	58	
Visceral	38	20	202	62	
**Tumor size**					
<5 cm	57	30	257	69	0.026
5–10 cm	73	38	183	54	
>10 cm	61	32	127	48	
Missing	2	1			
**Malignancy grade** **FNCLCC**					
1	61	28	316	81	<0.001
2	98	39	173	55	
3	90	33	103	36	
**Surgical margins**					
Wide	97	50	254	66	<0.001
Non-wide	96	50	128	46	
**Chemotherapy**					
No	156	81	207	57	0.669
Yes	37	19	180	51	
**Radiotherapy**					
No	132	68	216	58	0.190
Yes	61	32	152	52	

Abbreviations: MF/MFT, malignant fibroblastic/myofibroblastic tumors; MPNST, malignant peripheral nerve sheath tumor; STS, soft tissue sarcomas; NR, not reached; NOS, non specified.

## Materials and Methods

### Patients and Clinical Samples

The National Cancer Data Inspection Board and The Regional Committee for Research Ethics (REK nord) approved the study. The material was collected from our approved biobank for paraffin embedded material and slides. The Regional Committee approved that written consent from the patients for their information to be stored in the hospital database and used for research was not needed because most of the material was more than 10 years old, and most of the patients being dead. The ethics committee specifically waived the need for consent. Data were analyzed anonymously.

Primary tumor tissues from patients diagnosed with STS at the University Hospital of North Norway (UNN) from 1973 to 2006 and the Hospitals of Arkhangelsk region, Russia, were used in this retrospective study. In total, 496 potentially suitable patient records were identified from the hospitals databases. Of these, 247 patients were excluded due to missing clinical data (n = 86) or inadequate material for histological examination (n = 161). In addition 33 were excluded because of metastasis at the time of the diagnosis, 13 were excluded because they had no surgery, and 10 patients had both metastasis and no surgery. Eligible for this study were 193 patients. This report includes data for 131 Norwegian patients and 62 Russian patients followed until September 2009. The median follow-up was 38 (range 0–392) months. Complete demographic and clinical data were collected retrospectively. Formalin-fixed and paraffin-embedded tumor specimens were obtained from the archives of the Departments of Pathology at UNN and Archangelsk. The tumors were graded according to the French Fèdèration Nationales des Centres de Lutte Contre le Cancer (FNCLCC), [WHO Tumors of Soft Tissue and bone, 2002]. Wide resection margins were defined as wide local resection with free microscopic margins or amputation of the affected limb or organ. Non-wide resection margins were defined as either marginal or intralesional resection margins.

**Figure 2:**
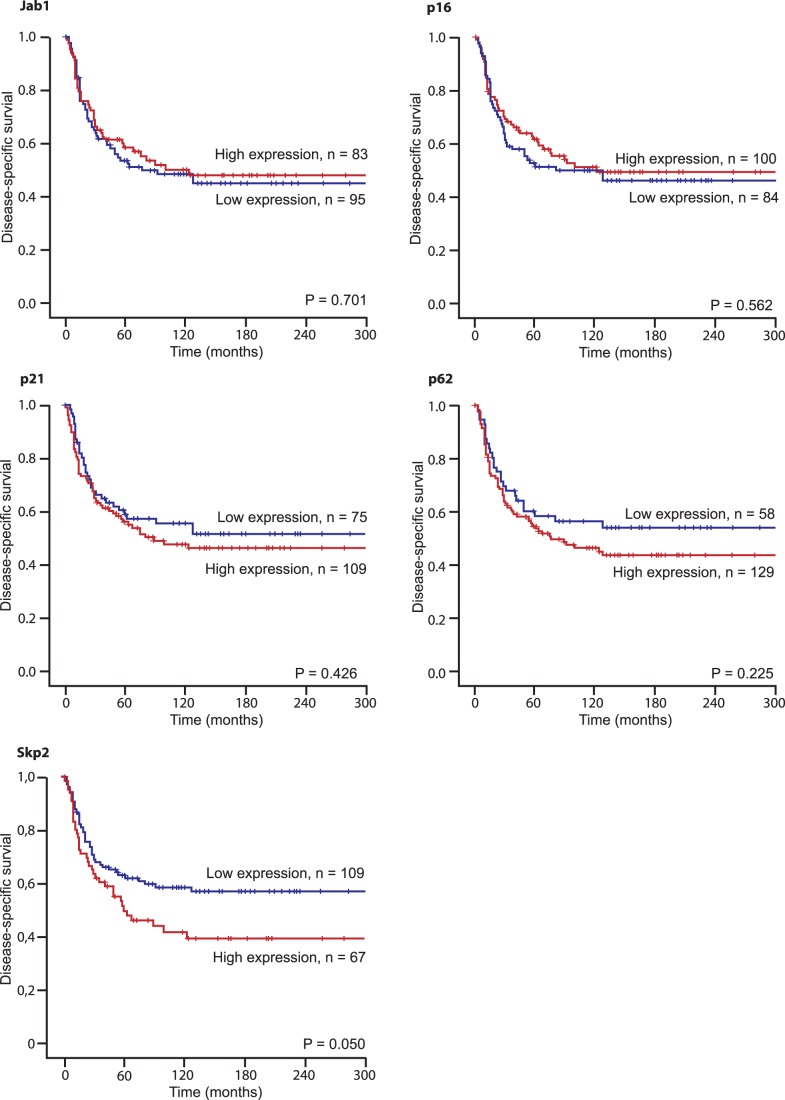
Disease-specific survival curves for high and low expression of different markers in patients with soft tissue sarcomas (N = 193).

**Table 2:** Expression of markers and their prediction for disease-specific survival in patients with soft tissue sarcomas (univariate analysis; log-rank test), N = 193.

Marker expression	Patients(n)	Patients(%)	Median survival(months)	5-Year survival(%)	P
**Jab1**					
Low	95	49	76	54	0.701
High	83	43	123	59	
Missing	15	8			
**p16**					
Low	84	13	80	51	0.562
High	100	52	123	62	
Missing	9	5			
**p21**					
Low	75	39	NR	59	0.426
High	109	56	89	56	
Missing	9	5			
**p62**					
Low	58	30	NR	59	0.255
High	129	67	76	55	
Missing	6	3			
**Ki67**					
Low	28	15	NR	68	0.007
Medium	56	29	NR	66	
High	99	51	57	48	
Missing	10	5			
**Skp2**					
Low	109	56	NR	63	0.050
High	67	45	59	50	
Missing	17	9			

Abbreviations: NR, not reached.

**Table 3:** Results of expression of p21, p62, Ki67 and Skp2 versus malignancy grade in patients with soft tissue sarcomas, N = 193.

Expression	Malignancy grade (%)						
	Grade 1	Grade 2	Grade 3	Total	Missing	Chi-Square	P-value
p21, Low	25 (33)	26 (35)	24 (32)	75	9	1.420	0.492
p21, High	28 (26)	45 (41)	36 (33)	109			
Total	53 (29)	71 (39)	60 (33)	184			
p62, Low	24 (41)	17 (29)	17 (29)	58	6	7.893	0.019
p62, High	28 (22)	55 (43)	46 (36)	129			
Total	52 (25)	72 (38)	63 (38)	187			
Ki67, Low	13 (46)	8 (29)	7 (25)	28	10	18.525	0.001
Ki67, Medium	22 (39)	22 (39)	12 (21)	56			
Ki67, High	15 (15)	42 (42)	42 (42)	99			
Total	50 (27)	72 (39)	61 (33)	183			
Skp2, Low	36 (33)	41 (38)	32 (29)	109	17	1.797	0.407
Skp2, High	16 (24)	27 (40)	24 (36)	67			
Total	52 (30)	68 (39)	56 (32)	176			

### Tissue Microarray Construction

The histology of all soft tissue sarcoma cases were reviewed by two pathologists (AV and SWS). Tissue microarrays (TMAs) were constructed for high-throughput molecular pathology research [33]. The most representative areas of viable tumor cells were carefully selected and marked on the hematoxylin and eosin (HE) slides for the corresponding donor blocks and sampled for the tissue microarray collector blocks. The TMAs were assembled using a tissue-arraying instrument (Beecher Instruments).

Studies suggest that punching multiple 0.6 mm cores from different regions captures the heterogeneity of the tumors more accurately than single 2 to 4 mm core [34]. Hence, we chose using two 0.6-mm cores of viable neoplastic tissue that were selected to be as representative as possible (different areas), after reviewing all original sections of the tumor and taking the heterogeneity in consideration. To include all core samples, 12 tissue array blocks were constructed. Multiple 4-µm sections were cut with a Micron microtome (HM355S) and stained by specific antibodies for immunohistochemistry (IHC).

**Table 4:** Correlation of marker expression in patients with soft tissue sarcomas (Pearson correlation), N = 193.

Markerexpression	Jab1	p16	p21	P62	Skp2	Ki67
**Jab1**	–	0.243**	0.372**	0.315**	0.305**	0.398**
**p16**	0.243*	–	0.208**	0.418**	0.275**	0.332**
**p21**	0.372**	0.208**	–	0.360**	0.312**	0.418**
**p62**	0.315**	0.418**	0.360**	–	0.195*	0.368**
**Skp2**	0.305**	0.275**	0.312**	0.195*	–	0.456**
**Ki67**	0.398**	0.332**	0.418**	0.368**	0.456**	–

* Correlation is significant at the 0.05 level (2 tailed).

** Correlation is significant at the 0.01 level (2 tailed).

### Immunohistochemistry (IHC)

All staining were performed in the Ventana Benchmark XT automated slide stainer (Ventana Medical System, Illkirch, France). Before staining the sections were incubated at 60 degrees Celsius over night. Tissue sections were incubated with primary mouse monoclonal antibodies recognizing Jab1 (Zymed, catalog number 18–7386, 1∶50), Skp2 (Zymed, catalog number 18–0307, 1∶10), p62 (BD Biosciences, catalog number 610832, 1∶50), Ki67 (Ventana, catalog number 790–4286, ready to use) and p21 (Dako, catalog number M7202, 1∶25), p16 (MTM lab, Germany, catalog number 9511, ready to use). We used a Ventana antibody diluent (catalog number 251-018). The incubation periods were 28 minutes for Jab1, 28 minutes for p16, 32 minutes for p62 and Ki67, 40 minutes for p21 and Skp2. This was followed by application of liquid diaminobenzidine as substrate-chromogen, yielding a brown reaction product at the site of the target antigen (Ventana iView DAB Detection Kit, catalog number 760–091). iVIEW DAB Detection Kit is an indirect biotin streptavidin system for detecting mouse and rabbit primary antibodies. The DAB chromogen produces a dark brown precipitate that is readily visualized by light microscopy. All reagents are provided pre-diluted by the manufacturer for use in Ventana Benchmark XT. Finally, slides were counterstained with hematoxylin to visualize the nuclei. For each antibody, including negative controls, all TMA staining were performed in a single experiment. In the TMA we also used cores from carcinomas and normal tissue as positive and negative controls.

**Figure 3:**
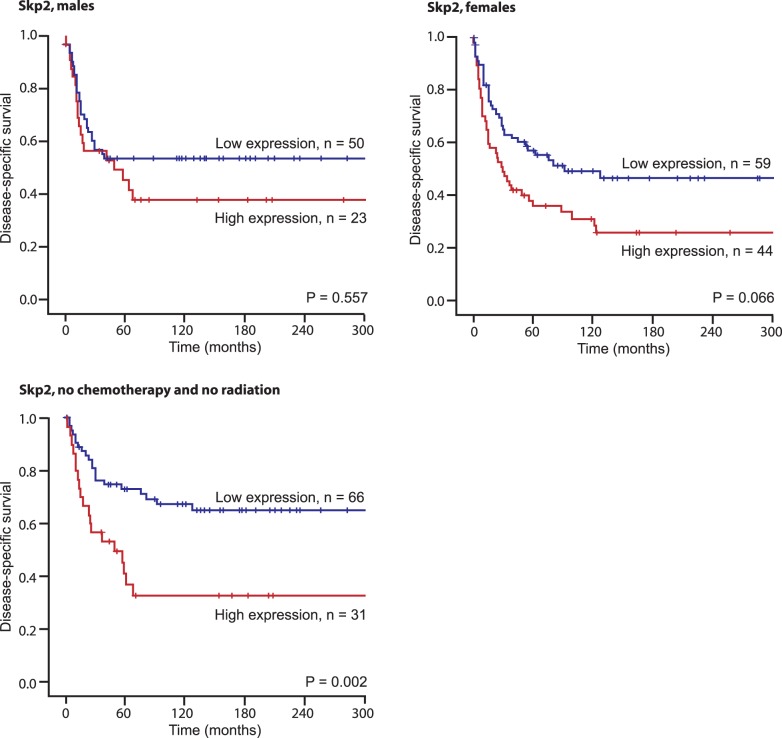
Disease-specific survival curves for high and low expression of Skp2 in males (N = 81), females (N = 112) and in patients not treated with chemotherapy or radiation (N = 104).

**Table 5:** Results of subgroup analysis of patients with expression of Skp2, N = 193.

Subgroup	Patients (n)	Patients (%)	Median survival (months)	5-Year survival (%)	P
**Age**					
0–60 years, Skp2 Low	52	53	NR	71	0.074
0–60 years, Skp2 High	38	38	67	56	
Missing	9	9			
>60 years, Skp2 Low	57	61	80	57	0.188
>60 years, Skp2 High	29	31	36	42	
Missing	8	9			
**Gender**					
Male, Skp2 Low	50	62	NR	63	0.577
Male, Skp2 High	23	28	67	61	
Missing	8	10			
Female, Skp2 Low	59	53	NR	63	0.066
Female, Skp2 High	44	39	49	44	
Missing	9	8			
**Nationality**					
Norwegian, Skp2 Low	67	51	NR	71	0.021
Norwegian, Skp2 High	57	44	89	54	
Missing	7	5			
Russian, Skp2 Low	42	68	91	51	0.177
Russian, Skp2 High	10	16	41	16	
Missing	10	16			
**Malignancy grade**					
1 or 2, Skp2 Low	77	59	NR	74	0.027
1 or 2, Skp2 High	43	33	89	56	
Missing	10	8			
3, Skp2 Low	32	51	26	37	0.970
3, Skp2 High	24	38	31	39	
Missing	7	11			
**Tumor size**					
**<**5 cm, Skp2 Low	37	63	NR	64	0.610
**<**5 cm, Skp2 High	17	29	NR	75	
Missing	5	8			
**>**5 cm, Skp2 Low	71	53	NR	63	0.018
**>**5 cm, Skp2 High	50	37	49	41	
Missing	13	10			
**Radiotherapy**					
No, Skp2 Low	77	58	NR	68	0.029
No, Skp2 High	45	34	58	46	
Missing	10	8			
Yes, Skp2 Low	32	52	62	53	0.744
Yes, Skp2 High	22	36	100	58	
Missing	7	11			
**Chemotherapy**					
No, Skp2 Low	94	60	NR	66	0.017
No, Skp2 High	49	31	58	45	
Missing	13	8			
Yes, Skp2 Low	15	41	45	47	0.743
Yes, Skp2 High	18	49	89	61	
Missing	4	11			

Abbreviations: NR, not reached.

**Table 6:** Results of Cox regression analysis summarizing prognostic factors in patients with soft tissue sarcomas.

	All patients, N = 193			No chemotherapy, no radiation, N = 104			Women, N = 112		
Factor	HazardRatio	95% CI	P	HazardRatio	95% CI	P	HazardRatio	95% CI	P
**Age**									
0–60 years	1.00			1.00			1.00		
>60 years	1.79	1.14–2.81	0.012	3.64	1.64–8.09	0.001	1.60	0.89–2.90	0.118
**Nationality**									
Norwegian	1.00			1.00			1.00		
Russian	1.49	0.89–2.48	0.129	1.67	0.72–3.86	0.232	2.33	1.17–4.66	0.016
**Tumor size**									
<5 cm	1.00		0.260*	1.00		0.963*	1.00		0.017*
5–10 cm	1.34	0.75–2.38	0.325	0.89	0.40–1.98	0.783	1.69	0.80–3.60	0.166
>10 cm	1.65	0.91–3.02	0.101	0.97	0.43–2.20	0.936	3.15	1.41–7.03	0.005
**Malignancy grade FNCLCC**									
1	1.00		<0.001*	1.00		0.004*	1.00		0.002*
2	2.70	1.37–5.32	0.004	2.36	1.04–5.34	0.040	4.79	1.93–11.88	0.001
3	4.86	2.45–9.65	<0.001	4.66	1.87–11.61	0.001	4.86	1.91–12.34	0.001
**Resection margins**									
Wide	1.00			1.00			1.00		
Non-wide	2.19	1.38–3.48	0.001	3.32	1.65–6.69	0.001	1.03	0.56–1.92	0.923
**Ki67**									
Low	1.00		0.392*	1.00		0.272*	1.00		0.214*
Medium	0.98	0.42–2.27	0.961	0.97	0.25–3.74	0.965	0.48	0.17–1.32	0.154
High	1.44	0.62–3.36	0.393	1.88	0.50–7.01	0.349	0.85	0.30–2.36	0.750
**Skp2**									
Low	1.00			1.00			1.00		
High	1.35	0–86−2.11	0.194	2.05	1.09–3.86	0.026	2.32	1.23–4.36	0.009

* Overall significance as a prognostic factor.

### Scoring of IHC

The ARIOL imaging system (Genetix, San Jose, CA) was used to scan the slides for antibody staining of the TMAs. The specimens were scanned at a low resolution (1.25×) and high resolution (20×) using an Olympus BX 61 microscope with an automated platform (Prior). The slides were loaded in the automated slide loader (Applied Imaging SL 50). In our experience it was difficult for the ARIOL imaging system to distinguish between tumor and stroma in soft tissue sarcomas. Representative and viable tissue sections were therefore scored manually on a computer screen semi-quantitatively for nuclear and/or cytoplasmic staining, Figure 1. The expression of Jab1, p16, p21, p62, Ki67 and Skp2 was scored as: 0, negative; 1, weak; 2, intermediate; 3, strong. The score for each patient was based on the mean scoring of cores from one or several biopsies. To achieve maximal reproducibility in all cases, every staining was dichotomised (low and high expression). Instead of using the overall mean score as cutoff, the cutoffs were chosen at levels securing statistically sufficient numbers in each group and appearing most biologically plausible. Hence, in this study the cutoff values varied among the different markers. High expression was defined as mean score >0 for p21 and Skp2, ≥0.33 for p62, ≥0.75 for p16 and ≥2.00 for Jab1 and Ki67. All samples were anonymized and independently scored by two pathologists (AV and SWS). In case of disagreement, the slides were re-examined and a consensus was reached by the observers. When assessing a variable for a given score, the scores of the other variables and the outcome were hidden from the observers.

### Statistical Methods

All statistical analyses were done using the statistical package SPSS (Chicago, IL), version 18. The IHC scores from each observer were compared for interobserver reliability by use of a two-way random effect model with absolute agreement definition. The intraclass correlation coefficient (reliability coefficient) was obtained from these results.

The Chi-square test and Fishers Exact test were used to examine the association between molecular marker expression and various clinicopathological parameters. Univariate analyses were done using the Kaplan-Meier method, and statistical significance between survival curves was assessed by the log rank test. Disease-specific survival (DSS) was determined from the date of histological-confirmed STS diagnosis. Correlation of marker expression was done using the Pearson correlation (2 tailed) at the 0.05 and the 0.01 level.

Multivariate analysis was carried out using the Cox proportional hazards model to assess the specific impact of each pre-treatment variable on survival in the presence of other variables. Only variables of significant value from the univariate analysis were entered into the Cox regression analysis. Probability for stepwise entry and removal was set at 0.05 and 0.10, respectively. The significance level used was P<0.05.

## Results

### Clinicopathological Variables

Demographic, clinical, and histopathological variables are shown in Table 1. Patient age ranged from 0–89 years (mean 55 years), and 42% of patients were male. The treatment option of choice was surgery (N = 193): 104 patients received surgery only; 52 patients received surgery and radiotherapy; 28 patients received surgery and chemotherapy; 9 patients received surgery, radiotherapy and chemotherapy. The 5-year survival for patients with wide and non-wide resection margins was 66% and 46% respectively, Table 1.

### Inter-observer Variability

There was good scoring reproducibility between the two investigating pathologists. Their scoring agreement was tested for p62 and Skp2. The IHC scores from each observer were compared using a two-way random effect model with absolute agreement definition. The intra-class correlation coefficients (reliability coefficients, r) obtained from these results were 0.82 for p62 (P<0.001) and 0.94 for Skp2 (P<0.001).

### Univariate Analyses

Nationality, histology, tumor size, malignancy grade and surgical margins were all significant indicators for disease-specific survival (DSS) in univariate analyses, Table 1.

In univariate analyses, increased expression of Ki67 (P = 0.007) and Skp2 (P = 0.050) correlated significantly with a shorter DSS, Table 2 and Figure 2. No such relationship was apparent for Jab1, p16, p21 and p62, but expression of p62 was positively correlated to malignancy grade (P = 0.019), Table 3. Ki67 was strongly positively correlated to malignancy grade (P = 0.001), Table 3.

High expression of the different markers was significantly correlated. There was weak (r = 0.20–0.29), moderate (r = 0.30–0.39) and strong (r = 0.40–0.69) positive correlations between the various examined markers. There was strong correlation between p16 and p62, strong correlation between Ki67 and p21/Skp2, moderate correlation between Jab1 and p21/p62/Skp2, moderate correlation between p21 and p62/Skp2 and moderate correlation between Ki67 and Jab1/p16/p62, Table 4.

In subgroup analyses (Table 5), increased Skp2 expression was associated with a shorter DSS in Norwegian patients (P = 0.021), those with malignancy grade 1 and 2 tumors (p = 0.027), tumors larger than 5 cm (P = 0.018), without radiotherapy (P = 0.029) and without chemotherapy (P = 0.017), Figure 3. There were no significant differences in the expression of the different immunomarkers in the different histological tumor groups (data not shown).

### Multivariate Analyses

Demographic, clinicopathological, and expression variables from the univariate analyses were entered into the multivariate Cox regression analysis (Table 6). In the multivariate analysis, age (P = 0.012), malignancy grade (P<0.001) and wide resection margins (P = 0.001) were independent prognostic factors for DSS. In addition, Skp2 had an independent prognostic impact in women (P = 0.009) and in patients not treated with chemotherapy or radiation (P = 0.026).

## Discussion

In this large scale study, we evaluated whether there is an association between tumor cell expression of Jab1, p16, p21, p62, Ki67 and Skp2 and survival in 193 non-GIST STS patients. Increased expression of Skp2 in patients with STS was an independent negative prognostic factor for DSS in women and in patients not administered chemotherapy or radiotherapy. To our knowledge, this is the first report where Skp2 is compared with Jab1, p16, p21 and p62 in STS and the first evidence of its possible clinical relevance in STS patients regarding potential benefits for adjuvant treatment with chemotherapy or radiation in the subgroup of patients with high expression of Skp2.

STS has different biological characteristics regardless of its histological phenotype. Its prognosis is in general poor, but also difficult to predict. In potentially curable STS prognostic markers should, ideally, guide further therapy following surgical resection. In our material, high expression of the different cell cycle control markers were significantly correlated. This is, however, expected since there is overlap in their mechanisms of action. Tsuchida et al. [35] suggested that Jab1 may play an important role in determining the differentiation stage of rhabdomyosarcoma cells by modulating the activity of CDK inhibitor p27. However, in our material, Jab1 showed no correlation with malignancy grade and had no prognostic impact on DSS in STS.

Epigenetic silencing of p16 might be critical early initiating events in the tumorigenesis of Ewing sarcoma family tumors [36]. p16 has been shown as a sensitive and specific marker for distinguishing atypical lipomatous tumor-well-differentiated liposarcoma and dedifferentiated liposarcoma from benign adipocytic neoplasms [37]. There is overexpression of p16 in uterine leiomyosarcoma compared to benign leiomyoma and normal myometrium [38]. p16 and pRb immunohistochemical expression increases with increasing tumour grade in mammary phyllodes tumours [39]. In a series of 38 pediatric osteosarcomas there was an inverse correlation between loss of pRB and p16 expression. Absence of p16 expression significantly correlated with decreased survival in univariate analysis [40]. Immunohistochemically decreased expressions of p16 was associated with poor prognosis in malignant peripheral nerve sheath tumor [41]. In a series of 21 uterine leiomyosarcomas, no statistically significant correlation between p16 expression and clinical stage, age, vascular space involvement, and recurrent disease could be found. Additionally, the overall survival did not significantly differ between p16-positive and p16-negative cases [42]. In a series of 84 uterine leiomyosarcomas, p16 did not show any significant correlation with survival [43]. Shim et al. [44] found no significant difference between the survival rate according to the p16 expression in 66 soft tissue sarcomas. In our material there was no correlation of p16 and malignancy grade or DSS.

Using *in vivo* RNA interference, Young et al. implicated the p53 target gene p21 as a critical mediator in sarcomagenesis [45]. The expression of p21 was closely associated with tumor malignancy grade, and therefore considered used as prognostic markers in a series of 152 STS [46]. López-Guerrero et al observed that the expression of p21 (P<0.015) was higher in disseminated than localized disease in patients with Ewing’s sarcoma tumors, but p21 did not influence progression free or overall survival [47]. In a series of 36 patients with leiomyosarcoma, p21 was not correlated with time to recurrence or overall survival [48]. Similarly, in our material p21 was not correlated to malignancy grade or DSS. This can be due to other bypass molecules involved in p53 suppression functions.

There are few publications regarding p62 and STS. Rolland et al. demonstrated that p62 expression in breast cancer is associated with tumor progression, but not DSS [49]. In a series of 109 non-small cell lung cancers, p62 were an independent factors predicting worse lung cancer-specific survival [50]. Kitamura et al. demonstrated cytosolic overexpression of p62 in prostate adenocarcinoma and high-grade PIN, suggesting that p62 might be a novel marker for prostatic malignancy [51]. However, in a series of 59 colorectal carcinomas, p62 had no prognostic value [52]. In our material, p62 correlated with malignancy grade, but not DSS.

High expression of Skp2 was reported to correlate with reduced overall survival in patients with myxofibrosacroma [53], [54]. Di Vizio et al. [55] found that Skp2 expression correlated with poor prognosis in gastrointestinal stromal tumors (GIST). Oliveira found that Skp2 expression is associated with cell proliferation and a worse prognosis in 182 soft tissue sarcomas [56]. In our material, high expression of Skp2 was a negative prognostic factor for DSS. Interestingly, this correlation was statistically significant in women only (P = 0.009), not men (P = 0.577). This may be related to differences in expression of sexual hormone receptors (ER and PGR) in male and female STS patients [57], [58]. An inverse correlation between Skp2 expression and the expression of ER and PGR has been reported by others investigating breast cancer [59] and other studies suggest that Skp2B may modulate the activity of the estrogen receptor [60], [61]. It should be a priority in further studies to explore the relations of Skp2, gender and DSS.

Since the regulation of p27Kip1 degradation is mediated by its specific ubiquitin ligase subunits S-phase kinase protein (Skp)2 and cyclin-dependent kinase subunit (Cks)1, many have an inverse correlation regarding overexpression of Skp2 and decreased expression of p27Kip1, an analysis of p27Kip1 and other Skp2 target proteins such as p53 and Rb would be helpful for substantiating the Skp2 observations. It has already been shown that Skp2 deficiency can enhance sensitivity of leukemia cells to chemotherapy [62] and Skp2 is itself being increasingly considered a possible target for breast cancer and prostate cancer therapy [63], [64]. Wang et al. found a significantly negative correlation between Skp2 expression and the survival of patients administered radiotherapy, indicating that overexpression of Skp2 was correlated with an increased radioresistance of esophageal squamous cell carcinoma. This is in contrast to our findings where chemotherapy and radiotherapy appear to reduce the negative survival impact in patients with Skp2 expressing STS tumors.

In conclusion, our data suggest that an increased Skp2 expression in women with STS was an independent indicator of a poor survival. Skp2 expression data may provide additional information to guide adjuvant therapy after surgical resection. Future studies are warranted to evaluate whether adjuvant chemotherapy or radiotherapy will improve the poor prognosis of Skp2 expressing soft tissue sarcoma patients.

## References

[1] Alamanda VK, Crosby SN, Archer KR, Song Y, Schwartz HS, et al. (2012) Primary excision compared with re-excision of extremity soft tissue sarcomas-is anything new? J Surg Oncol 105: 662–667. 10.1002/jso.23021 [doi]. 10.1002/jso.23021 22213171

[2] Grobmyer SR, Maki RG, Demetri GD, Mazumdar M, Riedel E, et al. (2004) Neo-adjuvant chemotherapy for primary high-grade extremity soft tissue sarcoma. Ann Oncol 15: 1667–1672. 15/11/1667 [pii];10.1093/annonc/mdh431 [doi]. 10.1093/annonc/mdh431 15520069

[3] Dickinson IC, Whitwell DJ, Battistuta D, Thompson B, Strobel N, et al (2006) Surgical margin and its influence on survival in soft tissue sarcoma. ANZ J Surg 76: 104–109. 16626341 10.1111/j.1445-2197.2006.03615.x

[4] Kiatisevi P, Asavamongkolkul A, Phimolsarnti R, Waikakul S, Benjarassamerote S (2006) The outcomes and prognostic factors of patients with soft-tissue sarcoma. J Med Assoc Thai 89: 334–342. 16696417

[5] Koea JB, Leung D, Lewis JJ, Brennan MF (2003) Histopathologic type: an independent prognostic factor in primary soft tissue sarcoma of the extremity? Ann Surg Oncol 10: 432–440. 12734093 10.1245/aso.2003.05.014

[6] Mendenhall WM, Zlotecki RA, Hochwald SN, Hemming AW, Grobmyer SR, et al (2005) Retroperitoneal soft tissue sarcoma. Cancer 104: 669–675. 16003776 10.1002/cncr.21264

[7] Raney RB Jr, Crist WM, Maurer HM, Foulkes MA (1983) Prognosis of children with soft tissue sarcoma who relapse after achieving a complete response. A report from the Intergroup Rhabdomyosarcoma Study I. Cancer 52: 44–50. 6850544 10.1002/1097-0142(19830701)52:1<44::aid-cncr2820520110>3.0.co;2-v

[8] Yang RS, Lane JM, Eilber FR, Dorey FJ, al Shaikh R, et al (1995) High grade soft tissue sarcoma of the flexor fossae. Size rather than compartmental status determine prognosis. Cancer 76: 1398–1405. 8620414 10.1002/1097-0142(19951015)76:8<1398::aid-cncr2820760815>3.0.co;2-b

[9] Zagars GK, Ballo MT, Pisters PW, Pollock RE, Patel SR, et al (2003) Prognostic factors for disease-specific survival after first relapse of soft-tissue sarcoma: analysis of 402 patients with disease relapse after initial conservative surgery and radiotherapy. Int J Radiat Oncol Biol Phys 57: 739–747. 14529779 10.1016/s0360-3016(03)00714-4

[10] Claret FX, Hibi M, Dhut S, Toda T, Karin M (1996) A new group of conserved coactivators that increase the specificity of AP-1 transcription factors. Nature 383: 453–457. 10.1038/383453a0 [doi]. 10.1038/383453a0 8837781

[11] Ahn J, Hong SA, Lee SE, Kim J, Oh YS, et al.. (2009) Cytoplasmic localization of Jab1 and p27 Kip1 might be associated with invasiveness of papillary thyroid carcinoma. Endocr J 56: 707–713. JST.JSTAGE/endocrj/K08E-372 [pii]. 10.1507/endocrj.k08e-372 19461157

[12] Tomoda K, Kubota Y, Kato J (1999) Degradation of the cyclin-dependent-kinase inhibitor p27Kip1 is instigated by Jab1. Nature 398: 160–165. 10.1038/18230 [doi]. 10.1038/18230 10086358

[13] Tomoda K, Kubota Y, Arata Y, Mori S, Maeda M, et al.. (2002) The cytoplasmic shuttling and subsequent degradation of p27Kip1 mediated by Jab1/CSN5 and the COP9 signalosome complex. J Biol Chem 277: 2302–2310. 10.1074/jbc.M104431200 [doi];M104431200 [pii]. 10.1074/jbc.M104431200 11704659

[14] Esteva FJ, Sahin AA, Rassidakis GZ, Yuan LX, Smith TL, et al (2003) Jun activation domain binding protein 1 expression is associated with low p27(Kip1)levels in node-negative breast cancer. Clin Cancer Res 9: 5652–5659. 14654548

[15] Goto A, Niki T, Moriyama S, Funata N, Moriyama H, et al.. (2004) Immunohistochemical study of Skp2 and Jab1, two key molecules in the degradation of P27, in lung adenocarcinoma. Pathol Int 54: 675–681. 10.1111/j.1440-1827.2004.01679.x [doi];PIN1679 [pii]. 10.1111/j.1440-1827.2004.01679.x 15363035

[16] Korbonits M, Chahal HS, Kaltsas G, Jordan S, Urmanova Y, et al (2002) Expression of phosphorylated p27(Kip1) protein and Jun activation domain-binding protein 1 in human pituitary tumors. J Clin Endocrinol Metab 87: 2635–2643. 12050228 10.1210/jcem.87.6.8517

[17] Li W, Sanki A, Karim RZ, Thompson JF, Soon LC, et al. (2006) The role of cell cycle regulatory proteins in the pathogenesis of melanoma. Pathology 38: 287–301. U0L232825J282126 [pii];10.1080/00313020600817951 [doi]. 10.1080/00313020600817951 16916716

[18] Kamb A, Gruis NA, Weaver-Feldhaus J, Liu Q, Harshman K, et al (1994) A cell cycle regulator potentially involved in genesis of many tumor types. Science 264: 436–440. 8153634 10.1126/science.8153634

[19] Caldas C, Hahn SA, da Costa LT, Redston MS, Schutte M, et al. (1994) Frequent somatic mutations and homozygous deletions of the p16 (MTS1) gene in pancreatic adenocarcinoma. Nat Genet 8: 27–32. 10.1038/ng0994-27 [doi]. 10.1038/ng0994-27 7726912

[20] Kim YT, Cho NH, Park SW, Kim JW (1998) Underexpression of cyclin-dependent kinase (CDK) inhibitors in cervical carcinoma. Gynecol Oncol 71: 38–45. S0090-8258(98)95134-4 [pii];10.1006/gyno.1998.5134 [doi]. 10.1006/gyno.1998.5134 9784316

[21] Mori T, Miura K, Aoki T, Nishihira T, Mori S, et al (1994) Frequent somatic mutation of the MTS1/CDK4I (multiple tumor suppressor/cyclin-dependent kinase 4 inhibitor) gene in esophageal squamous cell carcinoma. Cancer Res 54: 3396–3397. 8012957

[22] Rocco JW, Sidransky D (2001) p16(MTS-1/CDKN2/INK4a) in cancer progression. Exp Cell Res 264: 42–55. 10.1006/excr.2000.5149 [doi];S0014-4827(00)95149-8 [pii]. 10.1006/excr.2000.5149 11237522

[23] van de Putte G, Holm R, Lie AK, Trope CG, Kristensen GB (2003) Expression of p27, p21, and p16 protein in early squamous cervical cancer and its relation to prognosis. Gynecol Oncol 89: 140–147. S0090825803000106 [pii]. 10.1016/s0090-8258(03)00010-6 12694668

[24] Naini S, Etheridge KT, Adam SJ, Qualman SJ, Bentley RC, et al. (2008) Defining the cooperative genetic changes that temporally drive alveolar rhabdomyosarcoma. Cancer Res 68: 9583–9588. 68/23/9583 [pii];10.1158/0008-5472.CAN-07-6178 [doi]. 10.1158/0008-5472.CAN-07-6178 PMC2593800 19047133

[25] Nishijo K, Chen QR, Zhang L, McCleish AT, Rodriguez A, et al. (2009) Credentialing a preclinical mouse model of alveolar rhabdomyosarcoma. Cancer Res 69: 2902–2911. 69/7/2902 [pii];10.1158/0008-5472.CAN-08-3723 [doi]. 10.1158/0008-5472.CAN-08-3723 PMC2789740 19339268

[26] Dotto GP (2000) p21(WAF1/Cip1): more than a break to the cell cycle? Biochim Biophys Acta 1471: M43–M56. S0304-419X(00)00019-6 [pii]. 10.1016/s0304-419x(00)00019-6 10967424

[27] Brown DC, Gatter KC (1990) Monoclonal antibody Ki-67: its use in histopathology. Histopathology 17: 489–503. 2076881 10.1111/j.1365-2559.1990.tb00788.x

[28] Moscat J, Diaz-Meco MT, Wooten MW (2007) Signal integration and diversification through the p62 scaffold protein. Trends Biochem Sci 32: 95–100. S0968-0004(06)00327-6 [pii];10.1016/j.tibs.2006.12.002 [doi]. 10.1016/j.tibs.2006.12.002 17174552

[29] Duran A, Serrano M, Leitges M, Flores JM, Picard S, et al.. (2004) The atypical PKC-interacting protein p62 is an important mediator of RANK-activated osteoclastogenesis. Dev Cell 6: 303–309. S1534580703004039 [pii]. 10.1016/s1534-5807(03)00403-9 14960283

[30] Moscat J, Diaz-Meco MT, Albert A, Campuzano S (2006) Cell signaling and function organized by PB1 domain interactions. Mol Cell 23: 631–640. S1097-2765(06)00537-5 [pii];10.1016/j.molcel.2006.08.002 [doi]. 10.1016/j.molcel.2006.08.002 16949360

[31] Rodriguez A, Duran A, Selloum M, Champy MF, Diez-Guerra FJ, et al. (2006) Mature-onset obesity and insulin resistance in mice deficient in the signaling adapter p62. Cell Metab 3: 211–222. S1550-4131(06)00035-0 [pii];10.1016/j.cmet.2006.01.011 [doi]. 10.1016/j.cmet.2006.01.011 16517408

[32] Wang G, Chan CH, Gao Y, Lin HK (2011) Novel roles of Skp2 E3 ligase in cellular senescence, cancer progression, and metastasis. Chin J Cancer. cjc.011.10319 [pii];10.5732/cjc.011.10319 [doi]. 10.5732/cjc.011.10319 PMC3777478 22200179

[33] Nocito A, Kononen J, Kallioniemi OP, Sauter G (2001) Tissue microarrays (TMAs) for high-throughput molecular pathology research. Int J Cancer 94: 1–5. 11668471 10.1002/ijc.1385

[34] Kallioniemi OP, Wagner U, Kononen J, Sauter G (2001) Tissue microarray technology for high-throughput molecular profiling of cancer. Hum Mol Genet 10: 657–662. 11257096 10.1093/hmg/10.7.657

[35] Tsuchida R, Miyauchi J, Shen L, Takagi M, Tsunematsu Y, et al (2002) Expression of cyclin-dependent kinase inhibitor p27/Kip1 and AP-1 coactivator p38/Jab1 correlates with differentiation of embryonal rhabdomyosarcoma. Jpn J Cancer Res 93: 1000–1006. 12359053 10.1111/j.1349-7006.2002.tb02476.x PMC5927124

[36] von Levetzow C, Jiang X, Gwye Y, von Levetzow G, Hung L, et al. (2011) Modeling initiation of Ewing sarcoma in human neural crest cells. PLoS One 6: e19305. 10.1371/journal.pone.0019305 [doi];PONE-D-11-01315 [pii]. 10.1371/journal.pone.0019305 PMC3084816 21559395

[37] Thway K, Flora R, Shah C, Olmos D, Fisher C (2012) Diagnostic utility of p16, CDK4, and MDM2 as an immunohistochemical panel in distinguishing well-differentiated and dedifferentiated liposarcomas from other adipocytic tumors. Am J Surg Pathol 36: 462–469. 10.1097/PAS.0b013e3182417330 [doi]. 10.1097/PAS.0b013e3182417330 22301498

[38] Hakverdi S, Gungoren A, Yaldiz M, Hakverdi AU, Toprak S (2011) Immunohistochemical analysis of p16 expression in uterine smooth muscle tumors. Eur J Gynaecol Oncol 32: 513–515. 22053664

[39] Karim RZ, Gerega SK, Yang YH, Spillane A, Carmalt H, et al (2010) p16 and pRb immunohistochemical expression increases with increasing tumour grade in mammary phyllodes tumours. Histopathology 56: 868–875. 20497245 10.1111/j.1365-2559.2010.03562.x

[40] Maitra A, Roberts H, Weinberg AG, Geradts J (2001) AID-IJC1006>3.0.CO;2-V [pii].

[41] Endo M, Kobayashi C, Setsu N, Takahashi Y, Kohashi K, et al. (2011) Prognostic significance of p14ARF, p15INK4b, and p16INK4a inactivation in malignant peripheral nerve sheath tumors. Clin Cancer Res 17: 3771–3782. 1078-0432.CCR-10-2393 [pii];10.1158/1078-0432.CCR-10-2393 [doi]. 10.1158/1078-0432.CCR-10-2393 21262917

[42] Bodner-Adler B, Bodner K, Czerwenka K, Kimberger O, Leodolter S, et al. (2005) Expression of p16 protein in patients with uterine smooth muscle tumors: an immunohistochemical analysis. Gynecol Oncol 96: 62–66. S0090-8258(04)00742-5 [pii];10.1016/j.ygyno.2004.09.026 [doi]. 10.1016/j.ygyno.2004.09.026 15589581

[43] D’Angelo E, Espinosa I, Ali R, Gilks CB, Rijn M, et al. (2011) Uterine leiomyosarcomas: tumor size, mitotic index, and biomarkers Ki67, and Bcl-2 identify two groups with different prognosis. Gynecol Oncol 121: 328–333. S0090-8258(11)00065-5 [pii];10.1016/j.ygyno.2011.01.022 [doi]. 10.1016/j.ygyno.2011.01.022 21316747

[44] Shim BY, Yoo J, Lee YS, Hong YS, Kim HK, et al. (2010) Prognostic role of Rb, p16, Cyclin D1 proteins in soft tissue sarcomas. Cancer Res Treat 42: 144–150. 10.4143/crt.2010.42.3.144 [doi]. 10.4143/crt.2010.42.3.144 PMC2953777 20948919

[45] Young NP, Crowley D, Jacks T (2011) Uncoupling cancer mutations reveals critical timing of p53 loss in sarcomagenesis. Cancer Res 71: 4040–4047. 0008-5472.CAN-10-4563 [pii];10.1158/0008-5472.CAN-10-4563 [doi]. 10.1158/0008-5472.CAN-10-4563 PMC3160277 21512139

[46] Sabah M, Cummins R, Leader M, Kay E (2007) Immunoreactivity of p53, Mdm2, p21(WAF1/CIP1) Bcl-2, and Bax in soft tissue sarcomas: correlation with histologic grade. Appl Immunohistochem Mol Morphol 15: 64–69. 17536310 10.1097/01.pai.0000201809.43554.ed

[47] Lopez-Guerrero JA, Machado I, Scotlandi K, Noguera R, Pellin A, et al (2011) Clinicopathological significance of cell cycle regulation markers in a large series of genetically confirmed Ewing’s sarcoma family of tumors. Int J Cancer 128: 1139–1150. 20473914 10.1002/ijc.25424

[48] Leiser AL, Anderson SE, Nonaka D, Chuai S, Olshen AB, et al. (2006) Apoptotic and cell cycle regulatory markers in uterine leiomyosarcoma. Gynecol Oncol 101: 86–91. S0090-8258(05)00846-2 [pii];10.1016/j.ygyno.2005.09.055 [doi]. 10.1016/j.ygyno.2005.09.055 16289259

[49] Rolland P, Madjd Z, Durrant L, Ellis IO, Layfield R, et al. (2007) The ubiquitin-binding protein p62 is expressed in breast cancers showing features of aggressive disease. Endocr Relat Cancer 14: 73–80. 14/1/73 [pii];10.1677/erc.1.01312 [doi]. 10.1677/erc.1.01312 17395976

[50] Inoue D, Suzuki T, Mitsuishi Y, Miki Y, Suzuki S, et al. (2012) Accumulation of p62/SQSTM1 is associated with poor prognosis in patients with lung adenocarcinoma. Cancer Sci. 10.1111/j.1349-7006.2012.02216.x [doi]. 10.1111/j.1349-7006.2012.02216.x PMC7659245 22320446

[51] Kitamura H, Torigoe T, Asanuma H, Hisasue SI, Suzuki K, et al. (2006) Cytosolic overexpression of p62 sequestosome 1 in neoplastic prostate tissue. Histopathology 48: 157–161. HIS2313 [pii];10.1111/j.1365-2559.2005.02313.x [doi]. 10.1111/j.1365-2559.2005.02313.x 16405664

[52] Matsumoto K, Yamamoto J, Miura T (1993) Lack of prognostic value of immunoreactivity for p62 oncoprotein in colorectal carcinoma. Int J Colorectal Dis 8: 103–105. 8409682 10.1007/BF00299337

[53] Huang HY, Kang HY, Li CF, Eng HL, Chou SC, et al. (2006) Skp2 overexpression is highly representative of intrinsic biological aggressiveness and independently associated with poor prognosis in primary localized myxofibrosarcomas. Clin Cancer Res 12: 487–498. 12/2/487 [pii];10.1158/1078-0432.CCR-05-1497 [doi]. 10.1158/1078-0432.CCR-05-1497 16428491

[54] Huang HY, Huang WW, Wu JM, Huang CK, Wang JW, et al. (2008) Flow cytometric analysis of DNA ploidy and S-phase fraction in primary localized myxofibrosarcoma: correlations with clinicopathological factors, Skp2 expression, and patient survival. Ann Surg Oncol 15: 2239–2249. 10.1245/s10434-008-9968-0 [doi]. 10.1245/s10434-008-9968-0 18516647

[55] Di VD, Demichelis F, Simonetti S, Pettinato G, Terracciano L, et al. (2008) Skp2 expression is associated with high risk and elevated Ki67 expression in gastrointestinal stromal tumours. BMC Cancer 8: 134. 1471-2407-8-134 [pii];10.1186/1471-2407-8-134 [doi]. 10.1186/1471-2407-8-134 PMC2396636 18474118

[56] Oliveira AM, Okuno SH, Nascimento AG, Lloyd RV (2003) Skp2 protein expression in soft tissue sarcomas. J Clin Oncol 21: 722–727. 12586812 10.1200/JCO.2003.05.112

[57] Valkov A, Sorbye S, Kilvaer TK, Donnem T, Smeland E, et al. (2011) Estrogen receptor and progesterone receptor are prognostic factors in soft tissue sarcomas. Int J Oncol 38: 1031–1040. 10.3892/ijo.2011.920 [doi]. 10.3892/ijo.2011.920 21271213

[58] Valkov A, Kilvaer TK, Sorbye SW, Donnem T, Smeland E, et al. (2011) The prognostic impact of Akt isoforms, PI3K and PTEN related to female steroid hormone receptors in soft tissue sarcomas. J Transl Med 9: 200. 1479-5876-9-200 [pii];10.1186/1479-5876-9-200 [doi]. 10.1186/1479-5876-9-200 PMC3254077 22107784

[59] Davidovich S, Ben-Izhak O, Shapira M, Futerman B, Hershko DD (2008) Over-expression of Skp2 is associated with resistance to preoperative doxorubicin-based chemotherapy in primary breast cancer. Breast Cancer Res 10: R63. bcr2122 [pii];10.1186/bcr2122 [doi]. 10.1186/bcr2122 PMC2575536 18644126

[60] Bhatt S, Xiao Z, Meng Z, Katzenellenbogen BS (2012) Phosphorylation by p38 mitogen-activated protein kinase promotes estrogen receptor alpha turnover and functional activity via the SCF(Skp2) proteasomal complex. Mol Cell Biol 32: 1928–1943. MCB.06561-11 [pii];10.1128/MCB.06561-11 [doi]. 10.1128/MCB.06561-11 PMC3347406 22431515

[61] Umanskaya K, Radke S, Chander H, Monardo R, Xu X, et al. (2007) Skp2B stimulates mammary gland development by inhibiting REA, the repressor of the estrogen receptor. Mol Cell Biol 27: 7615–7622. MCB.01239-07 [pii];10.1128/MCB.01239-07 [doi]. 10.1128/MCB.01239-07 PMC2169057 17785450

[62] Wang J, Han F, Wu J, Lee SW, Chan CH, et al. (2011) The role of Skp2 in hematopoietic stem cell quiescence, pool size, and self-renewal. Blood 118: 5429–5438. blood-2010-10-312785 [pii];10.1182/blood-2010-10-312785 [doi]. 10.1182/blood-2010-10-312785 PMC3217347 21931116

[63] Wang Z, Gao D, Fukushima H, Inuzuka H, Liu P, et al. (2012) Skp2: a novel potential therapeutic target for prostate cancer. Biochim Biophys Acta 1825: 11–17. S0304-419X(11)00046-1 [pii];10.1016/j.bbcan.2011.09.002 [doi]. 10.1016/j.bbcan.2011.09.002 PMC3242930 21963805

[64] Wang Z, Fukushima H, Inuzuka H, Wan L, Liu P, et al. (2012) Skp2 is a promising therapeutic target in breast cancer. Front Oncol 1. 10.3389/fonc.2011.00057 [doi]. 10.3389/fonc.2011.00057 PMC3263529 22279619

